# Improving access to general practice for people with severe and multiple disadvantage: a realist review protocol (The Connection Study)

**DOI:** 10.12688/wellcomeopenres.19460.2

**Published:** 2023-10-11

**Authors:** Lucy Potter, Lorraine McDonagh, Jeremy Horwood, Michelle Farr, Gene Feder, Geoff Wong

**Affiliations:** 1 Centre for Academic Primary Care, University of Bristol, Bristol, England, BS8 2PN, UK; 2 Research Department of Primary Care and Population Health, University College London, London, England, NW3 2PF, UK; 3 The National Institute for Health and Care Research Applied Research Collaboration West (NIHR ARC West), University Hospitals Bristol and Weston NHS Foundation Trust, Bristol, BS1 2NT, UK; 4 Nuffield Department of Primary Care Health Sciences, Oxford University, Oxford, OX2 6GG, UK

**Keywords:** Inclusion Health, Access to healthcare, Inequalities, Realist review, Multiple disadvantage, Homelessness, Substance misuse

## Abstract

**Introduction:** Despite having high unmet health need, people with severe and multiple disadvantage (SMD, including combinations of homelessness, substance misuse, poor mental health and domestic violence and abuse) have poor access to general practice. This realist review will examine the existing evidence on interventions or aspects of routine care in general practice that are likely to increase or decrease access to general practice for people with SMD.

**Methods and analysis:** he aim of this review is to identify how these interventions or aspects of routine care increase or decrease access to general practice for people with SMD, in which contexts and for which patients. This review will involve a process comprising five sequential phases: (1) identifying established theories, (2) conducting an extensive search for proof, (3) selecting appropriate articles, (4) gathering and organising relevant data, and (5) utilising a realist analytical approach to synthesise evidence and make conclusions. Local implementation documents, in addition to published research studies, will be incorporated to enrich the analysis. We will collaborate with a stakeholder group consisting of people with lived experience of SMD and those who support them to advise us throughout.

**Ethics and dissemination:** Ethical approval is not required. Our findings will be disseminated through peer-reviewed publications, conference presentations and lay summaries and will be used to develop a complex intervention for improving access to general practice for and with people with severe and multiple disadvantage.

**PROSPERO registration number:** CRD42023390495

## Introduction

Severe and multiple disadvantage (SMD) is the experience of at least two of the four primary domains of disadvantage: homelessness, substance misuse, violence and abuse, and poor mental health ^1^. In England, 2.3 million adults (5.2% of the population) face two or more of these primary domains in a single year ^1^.

The combined and intersecting effect of multiple sources of severe disadvantage carries an extremely high burden of mortality, multi-morbidity and frailty ^2– 4^. Despite this need, people with SMD encounter significant barriers to accessing general practice and have lower patient enablement (the impact of the encounter on patients’ ability to cope with and understand their health problems) ^5– 7^. People with SMD are more likely to have negative experiences of healthcare, including stigma and discrimination, which can act as a lasting deterrent to help-seeking; appointment systems are often incompatible with their help seeking behaviours ^7, 8^. These patients are highly marginalised and the majority of general practice does not effectively include them ^7, 9– 11^.

Specialist homeless healthcare centres have emerged in most major cities in the UK to provide general practice to homeless people, but these cannot address the whole problem. They often have limited staffing and provision, many people experiencing SMD are not homeless, or may only be homeless temporarily, street sex workers often do not access homeless health due to safety concerns ^7^ and there are challenges in supporting people to transition from these services into mainstream care ^12^. Homeless health services or outreach that are separated from and not as comprehensive as mainstream general practice are not enough. There is a need for mainstream general practice to be more inclusive, integrated and accessible to marginalised patients.

General practice is a stretched system which can be challenging for marginalised patients to access, current provision is not proportionate to need ^6^. The concept of access in this study has four key aspects: availability (included direct and indirect costs to the patient), utilisation, service relevance and effectiveness, and equity (the extent to which resources are mobilised to reflect need) ^13^. Access to care is more than just being registered at a general practice; it requires the ‘human fit’ between the patient and healthcare staff and how care is delivered ^14^. People with SMD are more likely to have negative experiences of healthcare, including stigma and discrimination, which can act as a lasting deterrent to help seeking. Furthermore appointment systems are often incompatible with their help seeking behaviours ^7, 9^. Not being able to provide adequate care to patients experiencing SMD contributes to general practitioner (GP) stress and burnout ^6^. Understanding how to improve the ‘human fit’ between general practice and those most in need will benefit patients, staff and services. Mainstream and homeless general practice services are complex systems, the landscape of support organisations can be complex, patients with SMD can carry a history of complex experiences and needs. There is unlikely to be a simple solution to adequately improve access to general practice for people with SMD.

### Aim

The aim of the study is to identify effective interventions or routine care in general practice that can improve or decrease access to general practice for people with SMD in different settings- for whom, in what circumstances and how. This study also seeks to provide tailored preliminary recommendations based on the evidence gathered to enhance access to general practice for this group.

### Objectives

1. To conduct a realist review to enable understanding of what types of interventions or aspects of routine care in general practice can increase or decrease access to general practice for people with SMD in different settings, for whom, in what circumstances and how. We are particularly interested in modifiable causal or contextual factors which have the greatest scope to achieve the greatest benefit, and identifying the most important potential outcomes in improving access for people with SMD (relating to availability, utilisation, service relevance and effectiveness and equity).
2. To provide recommendations on tailoring, implementation and design strategies to improve access to general practice for people with SMD in different settings, for different groups.

### Research questions

How do interventions or aspects of routine care that improve or reduce access to general practice for people with severe and multiple disadvantage work, for whom and in what contexts?

1. What are the mechanisms by which interventions or aspects of routine care in general practice increase or decrease access to general practice for people with SMD?
2. What are the important contexts that influence intended and unintended outcomes?
3. What are important outcomes in improving access to general practice for people with SMD?
4. In what circumstances are interventions likely to be effective in improving access to general practice for people with SMD?

## Methods and analysis

**Figure 1.**
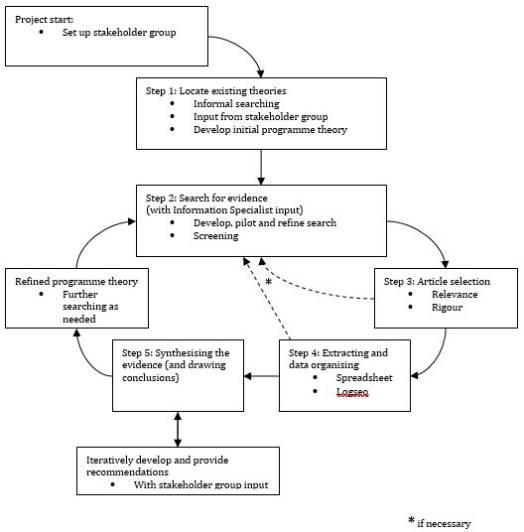
Steps of the realist review process. Adapted from Wong *et al.* ^17^ in accordance with the terms of the Creative Commons Attribution (CC BY 4.0).

This review will involve a process comprising Pawson *et al*’s five sequential phases of realist review: (1) identifying established theories, (2) conducting an extensive search for evidence, (3) selecting appropriate articles, (4) gathering and organising relevant data, and (5) utilising a realist analytical approach to synthesise evidence and make conclusions ^15^ (see Figure 1). A realist review approach is appropriate for these research questions as both general practice and access to services for people with SMD are complex; bringing them together requires a nuanced understanding of the mechanisms at play. General practice is complex because care delivery varies between practices and areas and people with SMD are not all the same. There may also be a range of outcomes related to improving access to general practice for marginalised patients ( *e.g.* health outcomes, patient and staff satisfaction, impact on future engagement) which are context specific and vary for different groups ( *e.g.* those who are receiving substance misuse treatment or not, those who are engaged with a local support organisation or not, those who are newly registering with a practice or have a pre-existing relationship with the staff/practice).

**Figure 2.**
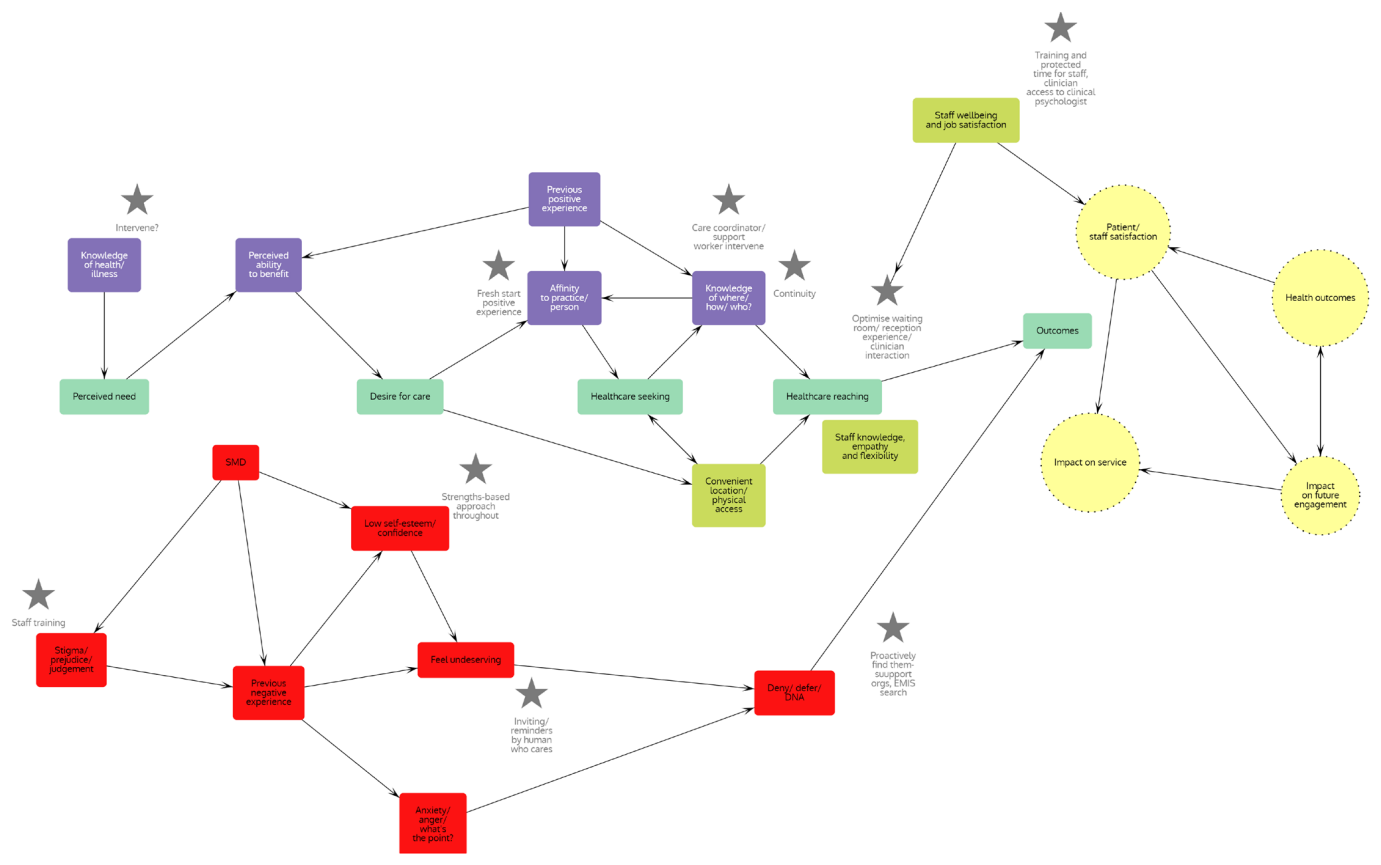
Initial programme theory of access to general practice for people with severe and multiple disadvantage (SMD). Developed using literature review, personal professional experience and co-production of service improvements with stakeholders (general practice and integrated care board (ICB) staff and people with lived experience of SMD). Main academic sources used- ^14, 18, 20^.

In order to effectively improve access to general practice, any analysis of evidence must consider the various contexts in which general practice is provided, including specialist outreach and homeless healthcare, and the different outcomes experienced by various groups. A realist review can provide the necessary understanding of how and why certain approaches work within specific contexts and outcomes, and offer recommendations for decision makers ^16^. This type of review draws on qualitative, quantitative, and mixed-methods research to develop theories about how contextual factors impact outcomes. Building explanations of how and why context can influence outcomes starts with the development and refinement of an initial programme theory of access to general practice for people with SMD. We have used substantive theory of access to general practice ^18^ and two realist reviews of which GW is a co-author - one of access to general practice for a different marginalised patient group (socioeconomically disadvantaged older people in rural areas) ^19^ and another of improving access of young adults with experience of homelessness to primary care dental services ^20^ and interrogated and adapted these with expert and lived experience input to develop an initial programme theory (see Figure 2). This initial programme theory will be further tested ( *i.e.* confirmed, refuted or refined) against empirical evidence during the review (see steps 2–5 in Figure 1) which are further detailed below. This review protocol has been registered with PROSPERO (registration number CRD42023390495) and will adhere to established quality and publication standards ^21^.

### Focus of the review

The focus of this review is to fill important knowledge gaps to move towards achieving more accessible general practice for people with SMD. To do this we will focus on which causal or contextual factors are modifiable, and have the greatest scope to achieve beneficial change ^22^. Additionally we will focus on potentially important outcomes in access to general practice for people with SMD, and how these relate to the components of access outlined above (availability, utilisation, service relevance and effectiveness and equity). Refining these points of focus will be achieved through iterative consultation of the literature and stakeholder and PPI groups.

Two realist reviews relevant to this topic, of which GW is a co-author, have been published ^19, 20^. The first focuses on access to general practice for a different but also highly marginalised patient group- socioeconomically disadvantaged older people in rural areas ^19^. The second on improving access to primary care dental services (rather than general practice) for young adults with experience of homelessness ^20^. While the focus populations of both of these are slightly different to our focus, interventions can sometimes benefit different marginalised patient groups ^23^; it is likely that there will be some transferable mechanisms and learnings from these reviews to ours ^24^. Additionally, we will focus on interventions developed in other services or with other marginalised groups that may be adapted to our context ^25^. Our intention is not to duplicate work but to build on the knowledge generated by previous research, and move towards the practical knowledge needed to develop or adapt a complex intervention to improve access to general practice for people with SMD.

### Patient and public involvement (PPI)/ stakeholders

We will consult with a wide range of stakeholders with professional and lived experience expertise and a variety of perspectives throughout the review. We are already collaborating with members of the public with lived experience of SMD, a co-production team called Bridging Gaps, and an organisation who supports them, One25, who we already have trusted relationships with ^26, 27^. We will invite policy-makers, commissioners and general practice staff to also participate in a stakeholder group. Depending on the preferences of participants we may join some of the Bridging Gaps and stakeholder meetings together or keep them separate. We will update and extend the membership of these groups as needed over the course of the review. As the programme theory is further refined throughout the review, the groups will be regularly consulted on the developing findings. LP will chair meetings with stakeholders (either face-to-face or online) four times during the review and communicate *via* telephone or email as needed. 

### Step 1: Locate existing theories

The goal of this step is to identify theories that explain how, for whom, why and in what circumstances interventions or aspects of routine care may increase or decrease access to general practice for people with SMD, and what patient and provider outcomes may result. The reasoning behind this step is that implicit or explicit theories of why particular factors are needed underpin the design of interventions or aspects of routine care. In order to achieve desired outcomes in improving access to general practice for people with SMD, we need to understand the details of what needs to be done to get there ^15^.

Our approach to developing an initial programme theory involves two iterative steps. We will consult with experts from our stakeholder groups and conduct initial informal searches of both grey literature and published research to identify existing theories that can improve access to general practice for people with SMD. This exploratory process will employ informal methods like snowballing and citation tracking ^28^, alongside more structured searching for theories ^29, 30^. We will use the insights gained from this process to develop an initial program theory for testing (confirm, refute or refine) in the review. We will refine this model within the project team and present it to our stakeholder groups for feedback.

### Step 2: Conduct formal search for evidence

The aim of this step is to locate a relevant ‘body of literature’ to further develop and refine the initial programme theory developed in step 1. LP will collaborate with an information specialist and the project team under the supervision of GW, who has extensive expertise in realist reviews to design, pilot, and conduct the search strategy. The formal database search will comprise electronic searches of MEDLINE (RRID:SCR_002185), EMBASE (RRID:SCR_001650), Web of Science (RRID:SCR_022706) and Scopus (RRID:SCR_022559) (detailed search strategies available on request). Additionally, LP will establish a regular search alert *via* Web of Science (RRID:SCR_022706) to remain updated with emerging literature as the review progresses.

We will also use grey literature to inform programme theory development. LP is a general practitioner experienced in providing general practice for patients with SMD and is part of Inclusion Health and Deep End GP clinical and research networks. Proposed strategies for identifying additional data, such as local implementation documents, include contacting relevant networks such the Deep End GP Network and the Faculty for Homeless and Inclusion Health to ask organisations and individuals to share relevant reports or evaluations. She will follow these relevant networks and experts in the field on Twitter/ X to remain updated with published and grey literature as it emerges. Additionally LP will contact leads in inequalities or Inclusion Health at NHS England, Office for Health Improvement and Disparities, the Health Foundation and the Kings Fund as well as searching their websites for relevant reports or evaluations. This list is not necessarily exhaustive.

***Screening***

We are using a broad search strategy including qualitative, quantitative, mixed-methods and relevant grey literature sources. The following criteria will be applied:

*Inclusion*

Intervention: Interventions or aspects of routine care aimed at (or potentially transferable to) improving access to general practice for people with SMD. Access to general practice defined using four key aspects- availability (included direct and indirect costs to the patient), utilisation, service relevance and effectiveness, and equity (the extent to which resources are mobilised to reflect need) ^13^.

Study design: all study designs.

Setting: Studies undertaken in high income countries, as defined by the World Bank will be included. All general practice settings including specialist primary healthcare provision such as homeless health services and outreach health services will be considered.

Participants: all adults (18 years and older)

Outcome measures: all outcome measures relevant to access to general practice for people with SMD.

*Exclusion*

Studies not undertaken in high-income countries, as defined by the World Bank, will be excluded. Editorials, opinion pieces, commentaries and drug effectiveness/ efficacy studies will be excluded. Screening will be undertaken by LP, based on title and abstract. LMD will independently review a 10% random sample of the citations retrieved from searching for quality control. If an abstract is not available, the complete text of a document will be referred to. Any discrepancies regarding inclusion will be settled through discussion. In case of persistent discrepancies, the matter will be referred to GW and resolved by majority vote.

***Additional searching***

The option of carrying out supplementary searches to gather more information to support the development of programme theory is an essential part of realist reviews. We may conduct additional searches, if required, to establish and evaluate specific aspects of our programme theory.

### Step 3: Article selection

Once initial screening is complete, LP will read the full text of any included articles. Inclusion will depend on the presence of relevant data that could inform program theory building or testing. While all articles that meet the inclusion criteria may not contain such information, those judged to be relevant (containing data that can contribute to programme theory development) and rigorous (using credible and trustworthy methods) according to Realist And Meta-narrative Evidence Syntheses: Evolving Standards (RAMESES) standards ^31^, will be included. Where appropriate, existing critical appraisal tools will be employed to evaluate data trustworthiness. By considering the role of every piece of retrieved data in refining or refuting our programme theory, even data of limited rigour may be incorporated ^32^. To verify consistent decision-making in article selection, a 10% random sample of relevant data will be evaluated and discussed by both LP and LMD. Any selection disagreements will be addressed by the resolution process described above in step 2, and the remaining 90% of article selection decisions will be made by LP (with the potential for project team discussion and joint reading when issues of relevance or rigour arise).

### Step 4: Extracting and organising data

LP will extract key characteristics (bibliographic information, study design, participants, settings and findings) of included papers into Microsoft Excel (RRID:SCR_016137). The full text of included papers will be uploaded into Logseq (data management software that allows enables coding and linked note taking). Relevant data from these papers will be coded in Logseq and understood as relevant to contexts, mechanisms and outcomes, or relationships between these. Coding will be deductive (generated from initial programme theory prior to data extraction), inductive (generated from data in included studies) and retroductive (generated by data interpretation, to deduce the mechanisms that lead to observed outcomes). Data extracted will be used to iteratively refine the theory if appropriate, and as this changes, included studies will be assessed again to search for relevant data to the revised theory. As with screening and inclusion decision-making, a 10% random sample of documents will be independently extracted, organised and discussed LP and by LMD with disagreements resolved with the processes described in step 2. Any disagreements will be addressed by the resolution process described in step 2, and the remaining 90% of extraction and data organising will be made by LP (with intermittent project team discussion of emerging key issues or controversies).

### Step 5: Synthesising the evidence and drawing conclusions

LP will use a realist logic of analysis that has been used in other realist reviews to interpret data included in the review ^33^ with support from the project team. As a part of our analysis and synthesis process, we will use a set of questions to evaluate the relevance and rigour of content within data sources and generate context-mechanism-output configurations (CMOCs) to refine the programme theory. These questions will include:

1. Is the section of this paper relevant to the development of the program theory?
2. Are the data trustworthy enough to warrant making changes to any portion of the program theory?
3. If the data is relevant and trustworthy enough, does it provide information that could be interpreted as context, mechanism, or outcome?
4. What is the CMOC (partial or complete) for the data? Are there further data to inform the particular CMOCs contained within this data? How does this CMOC relate to other CMOCs that have already been developed?
5. How does this particular (full or partial) CMOC relate to the program theory? Should the program theory be refined in light of this particular CMOC and any supporting data?
6. Has this CMOC or part of the program theory reached theoretical saturation?

We plan to gather information from multiple sources in order to better comprehend the connections between contexts, mechanisms, and outcomes. This will involve using data from various sources to create CMOCs. Due to the potential for missing components of these configurations within single data sources, it will be necessary to combine and compare information from multiple sources. We will compare between data sources to determine why certain outcomes have occurred and how context has played a role. We will use four methods of reasoning to interpret the data. These methods include:

1. Juxtaposing and comparing data from different sources
2. Investigating differences in seemingly similar conditions
3. Using methodological strength to determine the most reliable data when conflicting information arises
4. Generating explanations for differing outcomes in the same context.

From this refined programme theory we will formulate and assess recommendations and conclusions in collaboration with key stakeholders. It is likely that the refined programme theory at the end of the realist review will have knowledge gaps in it, which we are planning to address with an upcoming interview based realist evaluation. The refined programme theory from this realist review and the planned realist evaluation will be used to co-design a complex intervention to improve access to general practice for people with SMD.

## Conclusions

This realist review aims to deepen our comprehension of the various factors that either promote or hinder access to general practice for people with SMD. Those with lived experience of SMD will contribute to the refinement of the programme theory, analysis, interpretation, dissemination of findings and subsequent co-design of a complex intervention informed by this review. The quality and relevance of current literature in the field could limit the findings of this review. This review could offer insights into improving access to general practice for other marginalised groups as well as other services for people with SMD.

## Ethics and dissemination

Ethical approval is not required for this review. The stakeholders involved in this review will be involved in the study as PPI rather than included as research participants. Due to them being involved as part of the study team rather than used for data collection we do not require ethical approval, more detail can be found here. Our findings will be disseminated through peer-reviewed publications, conference presentations and user-friendly summaries and will be used to develop a complex intervention for improving access to general practice for and with people with severe and multiple disadvantage.

## Data Availability

No data or new software are associated with this article.
