# Supplementary material for: Improving access to general practice for people with severe and multiple disadvantage: a realist review protocol (The Connection Study)
Source: Wellcome Open Res. 2023 Oct 11;8:331. Originally published 2023 Aug 4. [Version 2] doi: 10.12688/wellcomeopenres.19460.2 (PMC10704067; doi:10.12688/wellcomeopenres.19460.2)
Supplement: Peer review history

# Reviewer report: Reviewer response for version 2

**DOI:** 10.21956/wellcomeopenres.22373.r68438

**Published:** 2023-12-07

**Authors:** Debi Bhattacharya

**Affiliations:** 1 School of Healthcare, University of Leicester, Leicester, England, UK

I approve the revisions.

Is the study design appropriate for the research question?

Yes

Is the rationale for, and objectives of, the study clearly described?

Yes

Are sufficient details of the methods provided to allow replication by others?

No

Are the datasets clearly presented in a useable and accessible format?

Partly

Reviewer Expertise:

NA

I confirm that I have read this submission and believe that I have an appropriate level of expertise to confirm that it is of an acceptable scientific standard.

# Reviewer report: Reviewer response for version 2

**DOI:** 10.21956/wellcomeopenres.22373.r68437

**Published:** 2023-11-22

**Authors:** David N. Blane

**Affiliations:** 1 General Practice and Primary Care, Institute of Health and Wellbeing, University of Glasgow, Glasgow, Scotland, UK

I have read through the responses and happy to approve.

Is the study design appropriate for the research question?

Yes

Is the rationale for, and objectives of, the study clearly described?

Yes

Are sufficient details of the methods provided to allow replication by others?

Partly

Are the datasets clearly presented in a useable and accessible format?

Not applicable

Reviewer Expertise:

NA

I confirm that I have read this submission and believe that I have an appropriate level of expertise to confirm that it is of an acceptable scientific standard.

# Reviewer report: Reviewer response for version 1

**DOI:** 10.21956/wellcomeopenres.21557.r64721

**Published:** 2023-09-19

**Authors:** David N. Blane

**Affiliations:** 1 General Practice and Primary Care, Institute of Health and Wellbeing, University of Glasgow, Glasgow, Scotland, UK

This is a protocol for a realist review/synthesis of existing evidence on interventions or aspects of routine care in general practice that are likely to increase or decrease access to general practice for people with SMD.

 I have two comments on this overall aim:

1. I think it would be simpler/‘cleaner’ to focus on *interventions* that have aimed to *improve *access, as I think it may be difficult to gather evidence on *aspects of routine care *that *decrease* access (and to determine the underlying mechanisms – what works, for whom, in what circumstances, and why – of these aspects of routine care), though I could be wrong.
2. The term “general practice” is used in the title and abstract, but “primary care” is used repeatedly throughout the rest of the protocol.  Will the focus be on general practice? And, if not, it is worth being clear on how primary care is being defined and which elements of primary care will be within scope (e.g. will you include dentistry, podiatry, district nursing, etc.?)

With regard to the conceptualisation of access, the authors outline different components of access that are of interest – namely, “availability, utilisation, service relevance and effectiveness and equity.” I am not convinced, however, that service *effectiveness* is really a component of access.  I can understand how *perceived relevance *of a service to an individual with SMD, for instance, may influence their likelihood of engaging with that service (this is very much in keeping with the notion of identification of *candidacy* for a service), but I think that exploring the *effectiveness *of said service is a much bigger – and quite separate – area of inquiry, particularly in the context of access to mainstream general practice.  Indeed, service effectiveness does not feature in other widely used theoretical frameworks of healthcare access, such as Levesque or the Dixon-Woods candidacy framework (a surprise omission from this paper, in my view).

 Objective 2 mentions “for different groups” but this does not feature in any of the RQs.

 The main RQ, as currently framed, does not make sense: “How do interventions or aspects of routine care improve or reduce access to general practice for people with severe and multiple disadvantage work, for whom and in what contexts?”

**Methods** – that authors state that the second phase of “Pawson’s five sequential phases of realist review” is “conducting an extensive search for proof”.  I would make two minor suggestions here: 1) change to “Pawson et al’s”, and 2) change “proof” to “evidence” (also in the Abstract).

 The authors go on to describe five steps which are slightly different from those of Pawson et al, which are:

1. Clarify scope
2. Search for evidence
3. Appraise primary studies and extract data
4. Synthesise evidence and draw conclusions
5. Disseminate, implement and evaluate.

Steps in the present paper are:

1. Locate existing theories
2. Conduct formal search for evidence
3. Article selection
4. Extracting and organising data
5. Synthesising the evidence and drawing conclusions.

These steps more closely follow Wong et al (as per Figure 1), but what I feel is missing from the paper is both the final step in Figure 1 (i.e. Refined programme theory – essentially, what you do once you have your CMOCs) and the final step described by Pawson et al (e.g. Draft and test out recommendations and conclusions with key stakeholders).  

 This could also be one of the four proposed meetings with stakeholders, which are otherwise kept fairly vague.

**Minor comments:**

 “ *Local implementation documents*” are mentioned as a source of evidence in the Abstract, but not in the main text (though perhaps this is the “grey literature” that is referred to in the Methods section).

 “There is not going to be one simple solution to solve all.”  I wonder if “There is unlikely to be a “one-size-fits-all” solution to improving access to primary care [or general practice] for people with SMD.”

Is the study design appropriate for the research question?

Yes

Is the rationale for, and objectives of, the study clearly described?

Yes

Are sufficient details of the methods provided to allow replication by others?

Partly

Are the datasets clearly presented in a useable and accessible format?

Not applicable

Reviewer Expertise:

Academic GP with expertise in inclusion health and realist review methods.

I confirm that I have read this submission and believe that I have an appropriate level of expertise to confirm that it is of an acceptable scientific standard, however I have significant reservations, as outlined above.

# Response

**Published:** 2023-10-04

**Authors:** Lucy Potter

**Affiliations:** Centre for Academic Primary Care, University of Bristol, Bristol, England, UK

Thank you for your really constructive review.  In response to your comments on the overall aim-

1. We agree that it would be simpler to just focus on interventions that have aimed to improve access. However, we have decided to stick to our more ambitious aim as this offer the potential of more information to help refine our programme theory and ultimately develop a more informed/ refined complex intervention. Also, we may not find relevant data on aspects of routine care that decrease access but we would at least know this is the case which may be of value as it identifies a knowledge gap.
2. Thank you for highlighting this discrepancy. The focus will be on general practice and have replaced the terms accordingly.

Regarding the conceptualisation of access including “availability, utilisation, service relevance and effectiveness and equity” we take your point that some others haven’t included effectiveness as part of access. From our PPI meetings with people with lived experience of SMD, the past experiences of accessing healthcare and the perception of effectiveness (i.e. whether they felt the encounter actually helped them) does seem to be a particularly important aspect to access for this population- for people with so much going on in their lives the knowledge of whether seeking help is likely to help them or not seems to be very important.

 Comprehensively understanding effectiveness including outcome measures could absolutely be a whole other piece of work but we don’t wish to exclude it as an important component of access for this population.

 Regarding Objective 2- different groups refers to the ‘for whom’ question. E.g. for people experiencing domestic violence as part of their SMD intervention X seems to be improve Y by the mechanism Z (this may not be the case for people with SMD experiencing poor mental health and homelessness but not domestic violence).

 The main RQ was missing a word which meant it didn’t make sense, thank you for pointing this out, we have now corrected it to- “How do interventions or aspects of routine care that improve or reduce access to general practice for people with severe and multiple disadvantage work, for whom and in what contexts?”

 We’ve added the highlighted text to Step 2- “Proposed strategies for identifying additional data, such as local implementation documents, include contacting relevant networks such the Deep End GP Network and the Faculty for Homeless and Inclusion Health to ask organisations and individuals to share relevant reports or evaluations.

 Thank you for questioning the simple solution sentence, we have amended this to “There is unlikely to be a simple solution to adequately improve access to general practice for people with SMD.”

 Re: Pawson suggestions- thank you we have made these changes. The final step in figure 1 is included in step 5- we have written “As a part of our analysis and synthesis process, we will use a set of questions to evaluate the relevance and rigour of content within data sources and generate context-mechanism-output configurations (CMOCs) to refine the programme theory.”

 The refined programme theory at the end of the realist review will have knowledge gaps in it, which we are planning to 'plug' with an upcoming interview based realist evaluation. At the end of step 5 we have added “From this refined programme theory we will formulate and assess recommendations and conclusions in collaboration with key stakeholders. It is likely that the refined programme theory at the end of the realist review will have knowledge gaps in it, which we are planning to address with an upcoming interview based realist evaluation. The refined programme theory from this realist review and the planned realist evaluation will be used to co-design a complex intervention to improve access to general practice for people with SMD."

 Thank you again for your really constructive feedback, we think it has really helped us improve the work. We look forward to your response. 

 Yours Sincerely,  Lucy Potter and team

# Reviewer report: Reviewer response for version 1

**DOI:** 10.21956/wellcomeopenres.21557.r64720

**Published:** 2023-09-07

**Authors:** Debi Bhattacharya

**Affiliations:** 1 School of Healthcare, University of Leicester, Leicester, England, UK

**Overview**

 The protocol is well written: the rationale for the work is clearly articulated and supported by the evidence. The methods adhere to accepted processes and are applied appropriately to answer the research questions.

**Areas for refinement**

 There is insufficient information regarding the formal search to permit replication - the full list of data sources is not provided refers to 'will include' this I infer that the list is not exhaustive. There is also insufficient information regarding the content of the search strategy in the aforementioned databases.

**Data synthesis**

 CMOCs will be created - will these be progressed any further e.g. final programme theory?

Is the study design appropriate for the research question?

Yes

Is the rationale for, and objectives of, the study clearly described?

Yes

Are sufficient details of the methods provided to allow replication by others?

No

Are the datasets clearly presented in a useable and accessible format?

Partly

Reviewer Expertise:

Behavioural science applied to realist review methods.

I confirm that I have read this submission and believe that I have an appropriate level of expertise to confirm that it is of an acceptable scientific standard, however I have significant reservations, as outlined above.

# Response

**Published:** 2023-10-04

**Authors:** Lucy Potter

**Affiliations:** Centre for Academic Primary Care, University of Bristol, Bristol, England, UK

Thank you for your considered and informed review. 

 With regards to your comments on the search strategy we have clarified our language to “The formal search will comprise electronic searches of MEDLINE (RRID:SCR_002185), EMBASE (RRID:SCR_001650), Web of Science (RRID:SCR_022706) and Scopus (RRID:SCR_022559).” Wellcome Open does not host supplementary material to enable sharing of our detailed search strategies. Instead we have added “(detailed search strategies available on request)”

 In answer to your question "CMOCs will be created - will these be progressed any further e.g. final programme theory?"- yes. Under step 5 we have written “As a part of our analysis and synthesis process, we will use a set of questions to evaluate the relevance and rigour of content within data sources and generate context-mechanism-output configurations (CMOCs) to refine the programme theory.” We have also explained our next steps in more detail at the end of Step 5. 

 Again, thank you for your contribution which has helped us improve the work. We look forward to seeing your response. 

 Best wishes,  Lucy Potter and team
